# Hypermethylation and Downregulation of UTP6 Are Associated With Stemness Properties, Chemoradiotherapy Resistance, and Prognosis in Rectal Cancer: A Co-expression Network Analysis

**DOI:** 10.3389/fcell.2021.607782

**Published:** 2021-08-18

**Authors:** Yiyi Zhang, Qiao Gao, Yong Wu, Yong Peng, Jinfu Zhuang, Yuanfeng Yang, Weizhong Jiang, Xing Liu, Guoxian Guan

**Affiliations:** ^1^Department of Colorectal Surgery, The First Affiliated Hospital of Fujian Medical University, Fuzhou, China; ^2^Fujian Maternity and Child Health Hospital, Affiliated Hospital of Fujian Medical University, Fuzhou, China; ^3^Department of Colorectal Surgery, Fujian Medical University Union Hospital, Fuzhou, China

**Keywords:** rectal cancer, chemoradiotherapy, weighted gene co-expression network analysis, UTP6, prognosis

## Abstract

**Background:**

To identify the hub genes associated with chemoradiotherapy resistance in rectal cancer and explore the potential mechanism.

**Methods:**

Weighted gene co-expression network analysis (WGCNA) was performed to identify the gene modules correlated with the chemoradiotherapy resistance of rectal cancer.

**Results:**

The mRNA expression of 31 rectal cancer patients receiving preoperative chemoradiotherapy was described in our previous study. Through WGCNA, we demonstrated that the chemoradiotherapy resistance modules were enriched for translation, DNA replication, and the androgen receptor signaling pathway. Additionally, we identified and validated UTP6 as a new effective predictor for chemoradiotherapy sensitivity and a prognostic factor for the survival of colorectal cancer patients using our data and the GSE35452 dataset. Low UTP6 expression was correlated with significantly worse disease-free survival (DFS), overall survival (OS), and event- and relapse-free survival both in our data and the R2 Platform. Moreover, we verified the UTP6 expression in 125 locally advanced rectal cancer (LARC) patients samples by immunohistochemical analysis. The results demonstrated that low UTP6 expression was associated with worse DFS and OS by Kaplan-Meier and COX regression model analyses. Gene set enrichment and co-expression analyses showed that the mechanism of the UTP6-mediated chemoradiotherapy resistance may involve the regulation of FOXK2 expression by transcription factor pathways.

**Conclusion:**

Low expression of the UTP6 was found to be associated with chemoradiotherapy resistance and the prognosis of colorectal cancer possibly via regulating FOXK2 expression by transcription factor pathways.

## Introduction

Preoperative chemoradiotherapy (CRT) and radical surgery have become the standard of care for stage II/III rectal cancer patients (Heald et al., 1982). The benefits of this multimodality therapy have been well documented, including tumor downsizing and downstaging, increased radical resection, and reduced local recurrence (Sauer et al., 2004, 2012; van Gijn et al., 2011). However, the treatment may increase the perioperative mortality rates by 48% (Rahbari et al., 2013). Moreover, 15% to 45% of the rectal cancers would are estimated to develop resistance to CRT, and might be exposed to CRT-related toxicitiestoxicity without oncological benefit (Ha et al., 2017). Therefore, the identification of valid biomarkers for colorectal cancer (CRC) with resistance to CRT has become imperative.

Currently, high-throughput sequencing is commonly used to screen and identify differentially expressed genes (DEGs). Conventional molecular biology methodology evaluates the variations in and functions of genes independently. Weighted gene co-expression network analysis (WGCNA) is an unbiased systematic biological approach, which clarifies the transcriptome function at a system-level, determines gene-gene correlations, and identifies gene modules with a high correlation across microarray data. It also bridges the gap between individual genes and tumorigenesis and progression (Zhang and Horvath, 2005; Langfelder and Horvath, 2008; Tian et al., 2017). WGCNA can facilitate the network-based gene screening approaches that screen key biomarkers associated with clinical traits in various cancers. However, this efficient bioinformatics approach has not yet been adopted to explore network-centric genes associated with CRT resistance in rectal cancer patients.

In this context, this study aimed to screen the relevant hub genes for CRT in our expression profile using WGCNA. Then, the hub genes were verified using testing data sets and patient tissue samples.

## Materials and Methods

### Subjects and Collection

In total, 31 locally advanced rectal cancer (LARC) patients receiving preoperative CRT between March 2016 to December 2016 in Fujian Medical University Union Hospital, China were enrolled as the internal dataset in this study, which was used for the gene-chip analysis. The CRT, treatment, and follow-up protocol were described in a previous study (Zhang et al., 2017). All patients received preoperative CRT and underwent R0 resection of primary colorectal tumors after neoadjuvant CRT. And the samples were collected at diagnosis and without any treatment before biopsy from colonoscopy. The chemoradiotherapy protocol was as follows: preoperative radiotherapy consisted of 45 Gy to the pelvis for 5 weeks (180 cGy/25 fractions) and a tumor boost of 5.4 Gy. Concomitant chemotherapy was administered with oral capecitabine (825 mg/m^2^ twice daily from day 1 to day 14 per cycle).

Moreover, to further validate the UTP6 expression in the LARC patients’ colonoscopic samples before CRT. Consecutive LARC patients who underwent CRT and radical resection between 2011 and 2014 were identified. A total of 125 patients were enrolled in the present study. This study was approved by the Institutional Review Board of Fujian Medical University Union Hospital (2013051).

### RNA Extraction, Quality Control, Labeling, Array Hybridization, and Data Analysis

Total RNA was extracted from the tissue mentioned previously using Trizol reagent (Invitrogen), according to the manufacturer’s protocol. RNA quantity and quality were measured by a NanoDrop ND-1000 and RNA integrity was assessed by standard denatured agarose gel electrophoresis. Sample labeling and array hybridization were performed according to the human GeneChip Analysis protocol (Affymetrix, Santa Clara, CA). The scanned data obtained from each microarray were normalized to correct for small differences in the amounts of each of the cRNA probes applied to the microarray and were processed for signal values using Affymetrix software (LIMS 5.0). The differentially expressed mRNAs were identified through fold-change filtering. Hierarchical clustering was performed using Agilent Gene Spring GX software (version 11.5.1). The upregulated and downregulated mRNA groups were defined as the mRNA expression in the CRT-responsive group compared with the CRT-non-responsive group. The Gene Ontology (GO) functional analysis and Kyoto Encyclopedia of Genes and Genomes (KEGG) pathway analysis were performed using standard enrichment computation method.

### Data Collection From the Gene Expression Omnibus and the Cancer Genome Atlas Database

**FIGURE 1:**
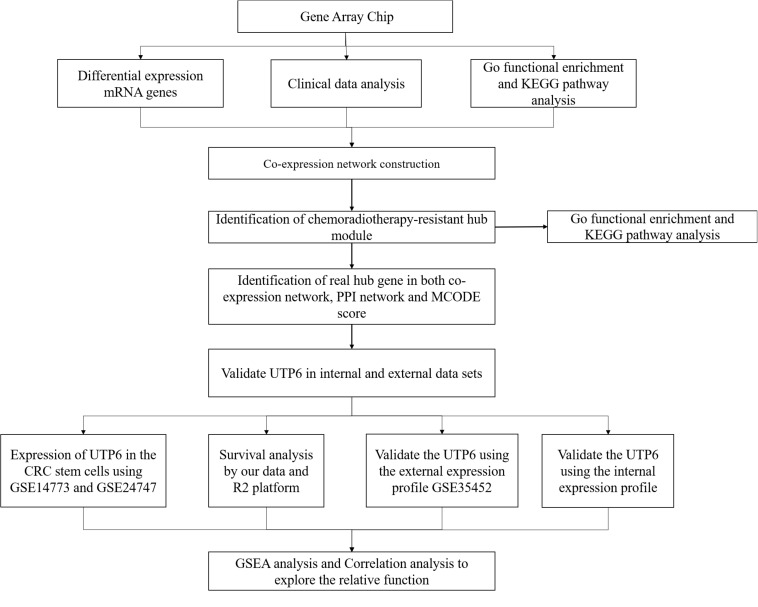
Workflow diagram of data preparation, processing, analysis, and validation in this study.

Datasets were downloaded from the Gene Expression Omnibus (GEO) database^1^ on December 19, 2019. The GSE35452 dataset included mRNA expression gene microarray of 46 rectal cancer patients receiving preoperative CRT and biopsy specimens were collected before preoperative CRT, which was used as the external validation dataset. Cancer stem cells are regarded as having self-renewal and differentiation properties and are one of the integral factors mediating the response to CRT (Baumann et al., 2008; Wahab et al., 2017). GSE14773 and GSE24747 were used to determine the hub gene expression in CRC stem cells. The GSE14773 dataset included one cell line with markers of CRC stem cells, high CD44, and CD166 expression, and the other one was a parental control CRC cell. The GSE24747 dataset included two groups, CD133 + and CD133-, CACO-2 cells. DNA methylation is considered an important component in gene expression regulation, and high levels of DNA promoter region methylation result in transcriptional silencing. Additionally, promoter regions methylation is reported to be associated with the CRT-resistance and considered as a predictor for CRT (Wilting and Dannenberg, 2012; Ivanova et al., 2013). The GSE104271 dataset was utilized to explore whether the hub genes were regulated by DNA methylation. As described by Huang et al. (2018) previously, FIBP knockdown cell lines were defined as cancer cells compared to cancer stem cells. The methylation data of the CRT-resistant genes CRC and normal colon tissues were obtained from The Cancer Genome Atlas (TCGA) in the UALCAN database^2^ (Chandrashekar et al., 2017). A flow diagram of the present study is shown in Figure 1.

### Definitions

Tumor response to CRT was graded according to the American Joint Committee on Cancer pathological tumor regression grade (AJCC TRG) (Amin et al., 2017; Benson et al., 2018); that is, TRG 0, no residual tumor cells; TRG 1, single cells or small groups of cells; TRG 2, residual cancer with the desmoplastic response; and TRG 3, minimal evidence of tumor response. Pathological complete response (pCR) was defined as the absence of viable tumor cells in the resected specimen, either at the primary site or in the lymph nodes. Venous blood samples were obtained within 1 week before CRT.

### Co-expression Network Construction

The WGCNA algorithm was previously described in detail (Zhang and Horvath, 2005). Briefly, first, we identified the qualification of the profile data. The co-expression network was constructed using the “WGCNA” package in R software (Horvath and Dong, 2008; Mason et al., 2009). Next, the correlation matrix was established and the soft threshold power was determined by analysis of the network topology. Then, the topological overlap matrix (TOM) was established (Yip and Horvath, 2007; Ivanova et al., 2013; Botía et al., 2017). Based on the phenotypic data of the groups, we calculated each module *p*-value by the *t*-test gene significance.

### CRT-Resistance Modules and Hub Gene Identification

To explore the relevant modules, we examined the association between module eigengenes (MEs) and CRT-resistance using Pearson’s correlation analysis. To identify the hub genes, the CRT-resistance model with the highest correlation coefficient (*P* < 0.05) in the data set was chosen, and the module also had the highest specific weight of all of the modules. The hub genes in the module were defined by module connectivity as measured by thean absolute value of the >0.3 in Pearson’s correlation >0.3 analysis. The protein–protein interaction (PPI) network was constructed by all genes in the CRT-resistance module, nd PPI network analysis was performed to screen the hub genes by Cytoscape. And the top 10 values were included for further analysis.

Statistical analysis was performed using SPSS software (version. 23 SPSS Inc., Chicago, IL, United States) and R software (version. 3.4.1). The continuous variables were reported as means and standard deviation from the analysis of variance test. The survival outcomes were assessed using Kaplan-Meier and log-rank analyses. Receiver operating characteristic (ROC) curve analysis was performed. Finally, the prognostic significance of the hub genes in CRC patients was analyzed using the R2: Genomics Analysis and Visualization Platform,^3^ on January, 03, 2020. The optimal cutoff points for the expression of UTP6 and FTSJ3 were calculated and determined using Cut-off Finder^4^ Visit and download data, 2019.12.26). Budczies et al. (2012), a new bio-informatics tool for biomarker assessment and outcome-based cut-point optimization, which identified the cut-off with the minimum *p*-values from log-rank χ2 statistics in terms of disease-free survival (DFS). A *P*-value of <0.05 was considered statistically significant.

### Gene Set Enrichment Analysis and Co-expression Gene Analysis

To explore the potential function of UTP6 in LARC patients, gene set enrichment analysis (GSEA) was performed in the patients in our previous datasets. A *P*-value of <0.05 and an enrichment score (ES) of >0.3 were set as the cutoff criteria. To further explore the correlation between UTP6 and relevant genes in the GSEA results, we analyzed the co-expressed genes in the TCGA data in the UALCAN dataset (Visit and download data, 2020.02.01).

### Immunohistochemical Analysis of UTP6 in the LARC Patients’ Sample

The protein expression of UTP6 in specimens obtained before and after CRT in 125 LARC patients was assessed using the immunohistochemical streptavidin-biotin complex method (Zhang et al., 2018). Phosphate-buffered saline (PBS) was used as the negative control and the image of the positive control from GE Healthcare Life Sciences. Immunoreactivity was scored by semi-quantitative analysis, and the fields were randomly selected in five directions (up, center, down, left, and right) under high magnification (^∗^400). The color was determined based on the intensity score as follows: 0 (no staining), 1 (light yellow), 2 (brown), and 3 (deep brown). The percentage of positive cells was scored as 0 (<5%), 1 (5–25%), 2 (25–50%), 3 (50–75%), and 4 (>75%). The mean value was calculated for each case with the aforementioned scoring methods and the final score was obtained by multiplying these two scores. The score between 0 and 4 was defined as the low expression and >4 was defined as high expression. All analyses were performed in a double-blind manner.

## Results

### Cluster Analysis

**FIGURE 2:**
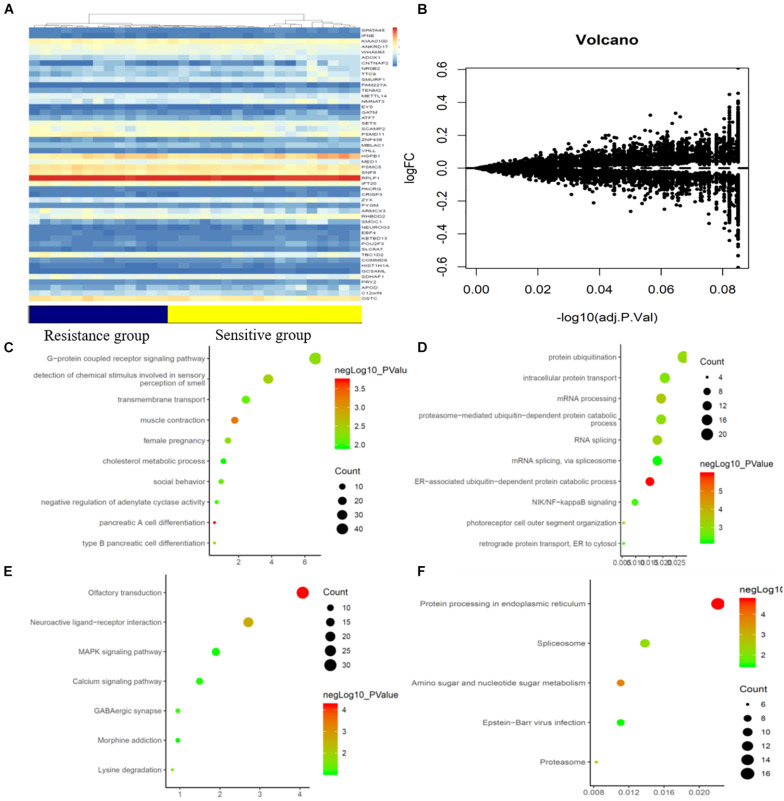
mRNAs expression profile comparison between chemotherapy-resistance and chemotherapy-sensitivity groups. Gene Ontology (GO) functional and Kyoto Encyclopedia of Genes and Genomes (KEGG) pathway analysis of the differentially expressed genes. **(A)** The hierarchical clustering of all targets values of mRNA expression profiling among samples. **(B)** Between the chemotherapy-resistance and chemotherapy-sensitivity group. The purple dots indicated the up-regulated genes of mRNAs and the green dots indicated the down-regulated genes of mRNAs. **(C)** GO functional analysis of the top ten functional classifications of the upregulated genes. **(D)** GO functional analysis of the top ten functional classifications of the downregulated genes. **(E)** KEGG pathway analysis of the top ten significant pathways of upregulated genes. **(F)** KEGG pathway analysis of the top ten pathways of downregulated genes.

A gene chip array was used to examine the gene expression profiles in primary tumor cells. A supervised hierarchical cluster analysis of the gene expression profiling data showed that the two groups had a clustering trend (Figures 2A,B). A total of 18419 genes were detected in the gene chip array. The SAM for the DEGs revealed that the two groups significantly differed in genes related to tumor cell biology, including 798 upregulated genes and 450 downregulated genes.

### GO Enrichment and KEGG Analysis

GO enrichment analysis was performed to investigate the molecular mechanism of the differently expressed genes involved in the resistance to CRT in LARC patients. We detected the top significant GO-enriched terms and KEGG terms in both the significantly upregulated and downregulated genes in rectal cancer patients (Figures 2C–F). The results showed that the top three significant GO terms for the upregulated genes were related to pancreatic A cell differentiation, muscle contraction, and type B pancreatic cell differentiation involved in the immune response. Among the downregulated genes, the top three significant GO terms were related to the ER-associated ubiquitin-dependent protein catabolic process, mRNA processing, and RNA splicing. Moreover, the top three significant KEGG terms in the upregulated genes were related to neuroactive ligand-receptor interaction, lysine degradation, and olfactory transduction involved in the immune response. In the downregulated genes, the top three significant GO terms were related to protein processing in the endoplasmic reticulum, amino sugar and nucleotide sugar metabolism, and the proteasome.

### Development of Weighted Co-expression Network and Identification of Key Modules

**FIGURE 3:**
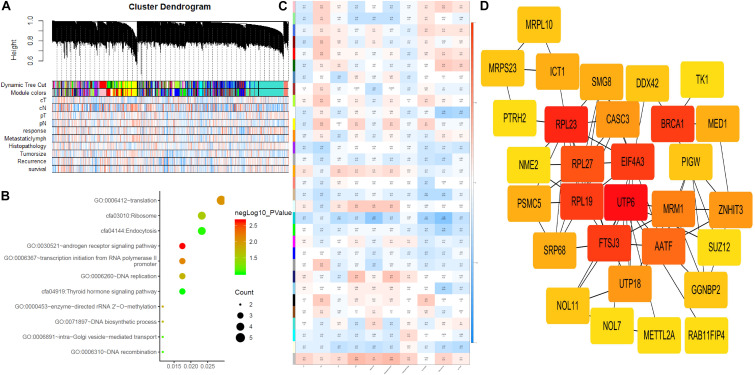
Weighted gene co-expression network analysis and hub gene screened. **(A)** Dendrogram of all expressed genes in the top 25% of variance clustered based on a dissimilarity measure (1-TOM). **(B)** KEGG pathway and GO functional analysis of the pathways of genes in darkturquoise modules. **(C)** Heatmap of the correlation between module eigengenes and CRT-resistance. **(D)** PPI network of genes that had the highest score in the PPI degree in the darkturquoise module. The color intensity in each node was proportional to the degree of connectivity in the weighted gene co-expression network.

To identify the hub genes, a weighted co-expression network was utilized to analyze our data (Figure 3A). A total of 31 modules were identified, as shown in Figure 3C. We further analyzed the relationship between CRT-resistance and the modules (Figure 3C). Among these modules, the module shown in dark turquoise (Figure 3C) had the strongest negative association with CRT-resistance (*r* = −0.45, *P* = 0.01), while the light cyan module showed the highest positive association with CRT-resistance (*r* = 0.25, *P* = 0.18). Then, we selected the dark turquoise module as the hub module. GO and KEGG enrichment analyses were conducted to explore the functions of the dark turquoise module. The results demonstrated that the dark turquoise module was mainly enriched for translation, DNA replication, and the androgen receptor signaling pathway (Figure 3B).

### Hub Gene Identification

Through co-expression analysis, 92 genes in the dark turquoise module were considered genes with high module connectivity. Then, the genes were analyzed by the PPI network and 59 genes in the dark turquoise module were identified as hub genes in the co-expression network. Ten hub genes were analyzed by the degree of PPI and the correlation between clinical traits and module connectivity was analyzed? (Figure 3D and Table 1). Finally, we chose the most associated genes, *UTP6* and *FTSJ3*, as the actual hub genes.

**TABLE 1:** Hub genes.

Genes	Clinical trait relationship (cor. geneTraitSignificance)	Degree in PPI network
UTP6	−0.43	12
RPL23	−0.23	11
RPL19	−0.23	10
EIF4A3	−0.21	10
FTSJ3	−0.50	10
BRCA1	−0.22	10
RPL27	−0.21	9
AATF	−0.19	8
MRM1	−0.33	7
ZNHIT3	−0.28	6

*PPI, protein–protein interaction.*

### Hub Gene Validation

**FIGURE 4:**
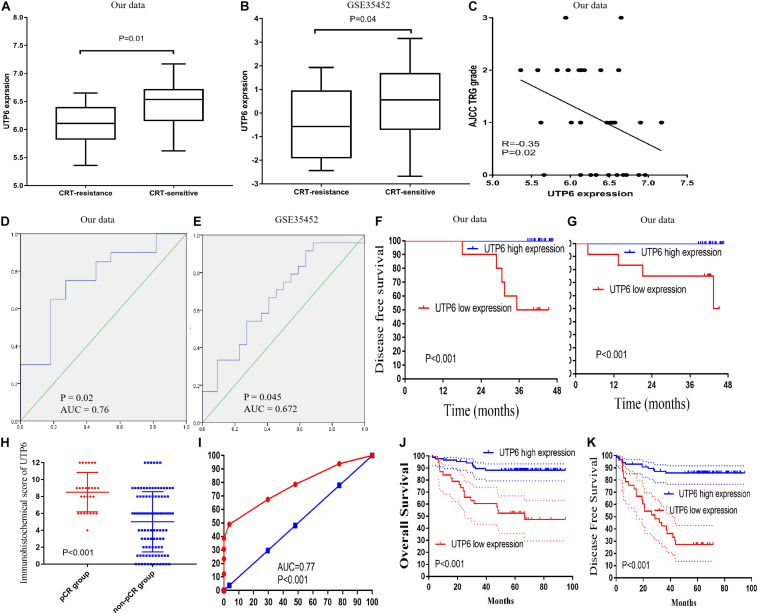
Validation of UTP6. **(A)** In our data (6.46 ± 0.40 vs. 6.07 ± 0.40, *P* = 0.02) and **(B)** GSE35452 (0.45 ± 1.51 vs. –0.47 ± 1.45, *P* = 0.042). **(C)** Pearson’s correlation analysis between the AJCC TRG grade and UTP6 expression. ROC curves and AUC statistics to evaluate the predictive efficiency of the UTP6 in our data and external data to distinguish CRT-resistance from CRT-sensitive CRC cases from **(D)** our data and **(E)** GSE35452. The disease-free survival **(F)** and overall survival **(G)** between low and high expression of UTP6. **(H)** UTP6 expression in the pCR and non-pCR group in the LARC patients’ sample. **(I)** ROC curves and AUC statistics to evaluate the predictive efficiency of the UTP6 in LARC patients’ samples to distinguish pCR from non-pCR LARC cases. The overall survival **(J)** and disease-free survival **(K)** between low and high expression of UTP6 in LARC patients’ sample.

To validate the hub genes, we examined the expression of UTP6 and FTSJ3 in rectal cancer tissues of CRT-resistant and CRT-sensitive cases in our datasets. In the internal testing data sets, the relative expression of UTP6 (6.46 ± 0.40 vs. 6.07 ± 0.40, *P* = 0.02; Figure 4A) and FTSJ3 (6.21 ± 0.32 vs. 5.75 ± 0.47, *P* < 0.01, Supplementary Figure 1A) was significantly increased in the CRT-sensitive tissues. The ROC curve demonstrated that both UTP6 (*P* = 0.02, AUC = 0.76, Figure 4D) and FTSJ3 (*P* < 0.01, AUC = 0.79, Supplementary Figure 1D) could efficiently discriminate CRT-resistant from CRT-sensitive rectal cancer cases. Moreover, Pearson’s analysis was conducted to determine whether the hub gene expression was associated with the TRG grade. The results demonstrated that UTP6 (*r* = −0.35, *P* = 0.02, Figure 4C) and FTSJ3 (*r* = −0.37, *P* = 0.04, Supplementary Figure 1C) had significant associations with the TRG grade.

We further explored the prognostic impact of UTP6 and FTSJ3 on the survival of LARC patients. Cut-off Finder was used to identify the cutoff values for UTP6 and FTSJ3. Cut-off Finder identified 6.18 and 5.97 as the cutoff values for UTP6 and FTSJ3 expression, respectively (Supplementary Figure 2). After a median follow-up period of 42 months (range, 4 – 47 months), low expressions of UTP6 and FTSJ3 were associated with significantly worse disease-free survival (DFS) compared to high expression (UTP6 low expression VS. high expression: 50.0% vs. 100.0%, *P* < 0.001; FTSJ3 low expression VS. high expression: 54.5% vs. 100.0%, *P* < 0.001), as shown in Figure 4F and Supplementary Figure 1F. Similarly, a high expression of UTP6 and FTSJ3 were correlated with better overall survival (OS) compared to low expression (UTP6 high expression VS. low expression: 100.0%, vs. 50.0%, *P* < 0.001; FTSJ3 high expression VS. low expression: 100.0% vs. 57.7%, *P* < 0.001), as demonstrated in Figure 4G and Supplementary Figure 1G.

### Hub Gene Validation in External Data

To validate the hub genes in our data set, we examined the expression level of UTP6 and FTSJ3 by comparing the rectal cancer tissues of CRT-resistant and-sensitive cases using an external dataset. In the external GSE35452 database, UTP6 expression was significantly higher in the CRT-sensitive tissues compared to the CRT-resistant tissues (0.45 ± 1.51 vs. −0.47 ± 1.45, *P* = 0.042; Figure 4B), but the expression of FTSJ3 was not statistically different between the two groups (0.11 ± 0.40 vs. −0.05 ± 0.53, *P* = 0.246; Supplementary Figure 1B). Thus, we chose UTP6 as the “real” hub gene for further analysis. ROC analysis revealed that UTP6 could efficiently discriminate CRT-resistant from CRT-sensitive cases (*P* = 0.045, AUC = 0.672; Figure 3E). However, FTSJ3 could not efficiently distinguish CRT-resistant from CRT-sensitive cases (*P* = 0.12, AUC = 0.63; Supplementary Figure 1D).

**FIGURE 5:**
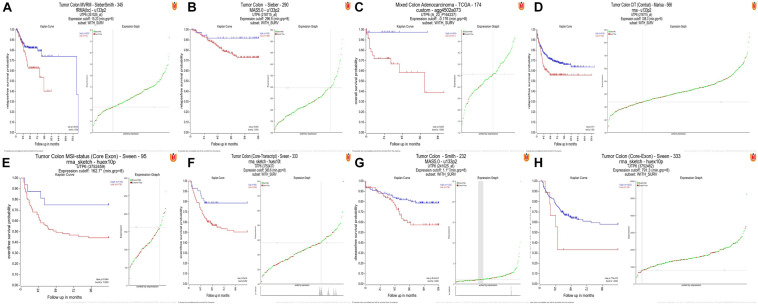
High UTP6 expression was associated with a better event-, disease-, and relapse-free survival. **(A–H)** High UTP6 expression was associated with a significantly better event-, disease- and relapse-free survival (both *P* < 0.05).

The R2 were utilized to plot Kaplan-Meier curves by using datasets “Tumor Colon-Sieber-290- MAS5.0-u133p2”, “Tumor Colon MVRM -SieberSmith-345- fRMA (bc) - u133p2”, “Tumor Colon-Smith-232-MAS5.0-u133p2”, “Tumor Colon (KRAS mut)-Hase-59- MAS5.0-u133p2”, “Tumor Colon CIT (Combat)-Marisa-566- rma-u133p2”, “Tumor Colon MSI-status (Core Exon)-Sveen-95-rma-sketch- huex10p”, “Mixed Colon Adenocarcinoma-TCGA-174 custom-agg4502a073”, “Tumor Colon (Core-Transcript)-Sveen-333-rma-sketch-huex10p” and “Tumor Colon (Core-Exon)-Sveen-333-rma- sketch-huex10p”. Low UTP6 expression was correlated with significantly worse event and relapse-free survival (all *P* < 0.05; Figures 5A–H).

### UTP6 Validation in LARC Patients’ Sample

To further verify UTP6 expression in the tumor samples, colonoscopy samples were collected from LARC patients before CRT. The immunohistochemical analysis demonstrated that higher UTP6 scores were associated with better prognosis in LARC patients following CRT. The 3-year OS rate of the low-UTP6 group was significantly lower than that in the high-UTP6 group (60.5% vs. 89.5%; *P* < 0.01, Figure 4J). Lower UTP6 scores were correlated with better DFS (Figure 4K). The 3-year DFS rate for the low-UTP6 group was significantly higher than that in the high-UTP6 group (42.4% vs. 87.1%; *P* < 0.01). Moreover, we compared the UTP6 expression in the pCR and non-pCR group. The results revealed that the relative expression of UTP6 was significantly increased in the pCR group (*P* < 0.01; Figure 4H). The ROC curve demonstrated that UTP6 could efficiently discriminate pCR from non-pCR LARC cases (*P* < 0.001, AUC = 0.77, Figure 4I). In addition, we detected the UTP6 expression in the rectal cancer tissues and adjuvant cancer tissues, the result demonstrated that UTP6 expression value was similar in rectal cancer and adjacent cancer tissues (*P* = 0.571, Supplementary Figure 3).

To explore the prognostic impact of UTP6 on the OS and DFS of LARC patients, a Cox regression analysis was performed. Univariate analysis revealed that tumor size (*P* = 0.004), ypTNM stage (*P* < 0.001), AJCC TRG grade (*P* = 0.013), UTP6 expression (*P* < 0.001), CD133 expression (*P* < 0.001), and the pre-CRT-CA19-9 level (*P* = 0.012) were independently associated with DFS in LARC patients following CRT and TME (Table 2). Multivariate Cox regression analysis demonstrated that UTP6 expression (HR = 0.398, 95%CI: 0.280 – 0.567, *P* < 0.001) and CD133 expression (HR = 1.205, 95%CI: 1.077 - 1.348, *P* = 0.001) were the independent predictors of DFS in LARC patients following CRT, as shown in Table 2.

**TABLE 2:** Cox regression analysis of predictive factors for disease-free survival in the training dataset patients with LARC following CRT (*n* = 125).

Variables	Univariate analysis			Multivariate analysis		
	HR	95% CI	*P*-value	HR	95% CI	*P*-value
Sex, male/female	0.961	0.504–1.833	0.905			
Age	1.002	0.976–1.027	0.904			
ASA	0.972	0.579–1.633	0.915			
Distance from the anal verge	0.976	0.872–1.093	0.677			
Tumor size	1.334	1.094–1.627	0.004	1.073	0.869–1.323	0.513
Pathological TNM stage	1.808	1.333–2.451	<0.001	1.260	0.846–1.878	0.256
AJCC grade	1.537	1.094–2.159	0.013	0.724	0.444–1.181	0.196
Interval time between CRT and surgery	0.982	0.860–1.121	0.785			
DRM involvement	5.137	0.683–38.652	0.112			
CRM involvement	4.226	0.573–31.150	0.157			
Pre-CRT cT stage	1.093	0.636–1.880	0.747			
Pre-CRT cN stage	1.073	0.382–3.011	0.893			
Organ preservation	1.386	0.583–3.297	0.460			
Pre-CRT CEA level	0.955	0.513–1.778	0.885			
Pre-CRT CA19-9 level	2.422	1.210–4.849	0.012	1.625	0.711–3.711	0.250
UTP6 expression	0.385	0.283–0.525	<0.001	0.730	0.650–0.820	<0.001
Postoperative complications	1.152	0.532–2.494	0.720			
Tumor differentiation	1.986	0.878–4.492	0.099			
Histopathology			0.425			
Expanding	Reference	Reference				
Infiltrating	0.678	0.163–2.820	0.593			
Ulcering	1.417	0.236–8.494	0.703			
CD133 expression	1.218	1.093–1.356	<0.001	1.205	1.077–1.348	0.001
FOXK2 expression	1.024	0.895–1.172	0.726			

*LARC, locally advanced rectal cancer; CRT, neoadjuvant chemoradiotherapy; HR, hazard ratio; CI, confidential interval; ASA, American Society of Anesthesiologists;*

*AJCC, American Joint Committee on Cancer; CEA, Carcinoembryonic Antigen; CRM, circumferential resection margin; DRM, distal resection margin.*

Upon the univariate analysis of the predictors of OS, tumor size (*P* = 0.006), ypTNM stage (*P* < 0.001), AJCC grade (*P* = 0.003), UTP6 expression (*P* < 0.001), the pre-CRT-CA19-9 level (*P* = 0.012), CD133 expression (*P* < 0.001), and tumor differentiation (*P* = 0.013) were independently associated with OS in LARC patients following CRT and TME (Table 3). The results from the multivariate Cox regression model demonstrated that UTP6 expression (HR = 0.398, 95%CI: 0.280 – 0.567, *P* < 0.001) and CD133 expression (HR = 1.185, 95%CI: 1.026 - 1.367, *P* = 0.021) were the independent predictors of OS in LARC patients following CRT, as shown in Table 3.

**TABLE 3:** Cox regression analysis of predictive factors for overall survival in the training dataset patients with LARC following CRT (*n* = 125).

Variables	Univariate analysis			Multivariate analysis		
	HR	95% CI	*P*-value	HR	95% CI	*P*-value
Sex, male/female	1.173	0.554–2.484	0.676			
Age	0.987	0.958–1.017	0.388			
ASA	0.915	0.483–1.733	0.785			
Distance from the anal verge	0.997	0.877–1.134	0.962			
Tumor size	1.394	1.102–1.764	0.006	1.071	0.795–1.442	0.653
Pathological TNM stage	1.934	1.379–2.713	<0.001	1.132	0.733–1.748	0.577
AJCC grade	1.869	1.236–2.827	0.003	1.073	0.614–1.874	0.805
Interval time between CRT and surgery	0.927	0.787–1.093	0.367			
DRM involvement	6.957	0.879–55.039	0.066			
CRM involvement	4.390	0.595–32.402	0.147			
Pre-CRT cT stage	0.804	0.431–1.500	0.492			
Pre-CRT cN stage	3.738	0.508–27.480	0.195			
Organ preservation	1.925	0.783–4.731	0.154			
Pre-CRT CEA level	1.109	0.533–2.306	0.782			
Pre-CRT CA19-9 level	2.738	1.245–6.021	0.012	1.578	0.538–4.625	0.406
UTP6 expression	0.507	0.356–0.723	<0.001	0.822	0.722–50.9	0.003
Postoperative complications	0.738	0.257–2.122	0.573			
Tumor differentiation	2.955	1.260–6.931	0.013	1.190	0.363–3.895	0.774
Histopathology			0.209			
Expanding	Reference	Reference				
Infiltrating	0.915	0.124–6.753	0.930			
Ulcering	2.699	0.281–25.961	0.390			
CD133 expression	1.248	1.097–1.421	0.001	1.185	1.026–1.367	0.021
FOXK2 expression	1.117	0.955–1.307	0.167			

*LARC, locally advanced rectal cancer; CRT, neoadjuvant chemoradiotherapy; HR, hazard ratio; CI, confidential interval; ASA, American Society of Anesthesiologists;*

*AJCC, American Joint Committee on Cancer; CEA, Carcinoembryonic Antigen; CRM, circumferential resection margin; DRM, distal resection margin.*

### Association of UTP6 Expression With Patient Characteristics and Perioperative Clinicopathological Parameters in the LARC Patients Following CRT

No significant differences were observed between UTP6 low expression group and UTP6 high expression group in terms of gender, age, American Society of Anesthesiologists (ASA) grade, interval time between CRT and surgery, distance from the anal verge, clinical T stage, clinical N stage, pre-CRT CEA level, and pre-CRT CA199 level, as shown in Table 4.

**TABLE 4:** Baseline characteristics in patients with LARC following CRT stratified by UTP6 expression (*n* = 125).

Characteristics	UTP6 low expression (*n* = 85)	UTP6 low expression (*n* = 40)	*P*-value
Sex (%)			0.421
Male	58 (68.2)	24 (60.0)	
Female	27 (31.8)	16 (40.0)	
Age (years)	58.3 ± 10.9	57.1 ± 14.9	0.620
ASA score (%)			0.980
1	59 (69.4)	28 (70.0)	
2	21 (24.7)	10 (25.0)	
3	5 (5.9)	2 (5.0)	
Distance from the anal verge (cm)	6.4 ± 3.0	6.7 ± 2.0	0.603
Interval time between CRT and surgery (weeks)	8.3 ± 2.5	8.4 ± 1.5	0.797
Pre-CRT cT stage (%)			0.236
T2	5 (5.9)	0 (0.0)	
T3	35 (41.2)	15 (37.5)	
T4	45 (52.9)	25 (62.5)	
Pre-CRT cN stage (%)			0.222
N0	11 (12.9)	2 (5.0)	
N +	74 (87.1)	38 (95.0)	
Pre-CRT CEA (%)			0.702
<5.0 ng/ml	50 (58.8)	22 (55.0)	
≥5.0 ng/ml	35 (41.2)	18 (45.0)	
Pre-CRT CA19-9 (%)			0.306
<37.0 ng/ml	73 (85.9)	31 (77.5)	
≥37.0 ng/ml	12 (14.1)	9 (22.5)	

*LARC, locally advanced rectal cancer; CRT, neoadjuvant chemoradiotherapy; ASA, American Society of Anesthesiologists; CEA, Carcino Embryonic Antigen.*

No significant differences were observed between UTP6 low expression group and UTP6 high expression group in terms of pathological type, postoperative complication, circumferential resection margin (CRM) involvement, tumor differentiation, perineural invasion, vascular invasion, and organ preservation procedure (Table 5). Compared to the UTP6 low expression group, the UTP6 high expression group was associated with an increased metastasis to the lymph nodes (0.45 ± 1.4 vs. 3.1 ± 7.1, *P* < 0.001), pathological T stage, pathological N stage (all *P* < 0.01), and poorer TRG grade (*P* < 0.01). In the training dataset, in the high-risk score group a larger tumor size was seen (2.7 ± 1.2 vs. 3.5 ± 1.6, *P* = 0.002), more lymph nodes were retrieved (11.2 ± 8.7 vs. 15.7 ± 13.8, *P* = 0.028), and poorer histopathology was observed (*P* = 0.026) compared with UTP6 low expression group.

**TABLE 5:** Operative and postoperative outcomes in patients with LARC following CRT stratified by UTP6 expression (*n* = 125).

Characteristics	UTP6 high expression (*n* = 85)	UTP6 low expression (*n* = 40)	*P-*value
Pathological type (%)			0.445
Ulcering	80 (94.1)	35 (8735)	
Expanding	3 (3.5)	3 (7.5)	
Infiltrating	2 (2.4)	2 (6.0)	
Histopathology (%)			0.026
Adenocarcinoma	80 (94.1)	32 (80.0)	
Mucinous or signet ring cell carcinoma	5 (5.9)	8 (20.0)	
Tumor differentiation (%)			0.077
Well to moderately differentiated	78 (91.8)	32 (80.0)	
Poorly differentiated and others	7 (8.2)	8 (20.0)	
Postoperative complications (%)	16 (18.8)	5 (12.5)	0.450
Organ preservation (%)	76 (89.4)	33 (82.5)	0.389
Lymph nodes retrieved	11.2 ± 8.7	15.7 ± 13.8	0.028
Metastatic lymph nodes	0.45 ± 1.4	3.1 ± 7.1	<0.001
CRM involvement (%)	1 (1.2)	2 (5.0)	0.240
Tumor size (cm)	2.7 ± 1.2	3.5 ± 1.6	0.002
Pathological T stage (%)			<0.001
0	27 (31.8)	0 (0.0)	
1	7 (8.2)	2 (5.0)	
2	15 (17.6)	10 (25.0)	
3	35 (41.2)	23 (57.5)	
4	1 (1.2)	5 (12.5)	
Pathological N stage (%)			0.001
0	72 (84.7)	22 (55.0)	
1	9 (10.6)	11 (27.5)	
2	4 (4.7)	7 (17.5)	
Pathological M stage (%)			0.036
0	84 (98.8)	36 (90.0)	
1	1 (1.2)	4 (10.0)	
TRG (%)			<0.001
0	27 (31.8)	0 (0.0)	
1	27 (31.8)	11 (27.5)	
2	25 (29.4)	23 (57.5)	
3	6 (7.1)	6 (15.0)	
Nerval invasion (%)	0 (0.0)	0 (0.0)	1.000
Vascular invasion (%)	2 (2.4)	0 (0.0)	1.000

*LARC, locally advanced rectal cancer; CRT, neoadjuvant chemoradiotherapy;*

*CRM, circumferential resection margin; DRM, distal resection margin; TRG, tumor regression grade; pCR: pathological complete response.*

### Analysis DNA Promoter Region Methylation of UTP6 in CRC Tissues and Expression, Promoter Methylation of UTP6 in CRC Stem Cells

**FIGURE 6:**
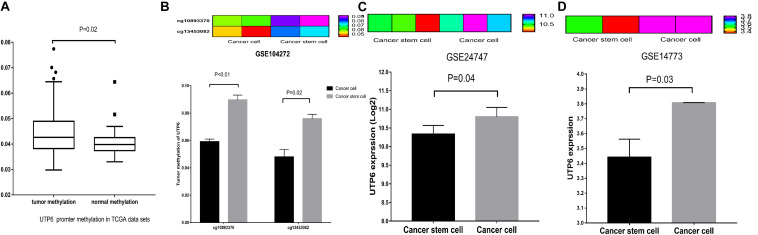
UTP6 had increased promoter hypermethylation in colon tumors, lower expression in CRC stem cells. **(A)** From the TCGA database. UTP6 had increased promoter hypermethylation in colon cancer. **(B)** UTP6 had higher promoter methylation levels in cg10893370 and cg13453082 sites in CRC stem cells. Heat map and bar graph showed that CRC non-stem cells had higher UTP6 expression compared with CRC stem cells in GSE24747 **(C)** and GSE14773 **(D)**.

To explore whether UTP6 was modulated by methylation, we evaluated data on the methylation of the *UTP6* promoter region in CRC and normal colon tissues. Based on the ULACAN database, methylation of the *UTP6* promoter was decreased in normal colon tissue compared to CRC tissue (0.0409 ± 0.0013 vs. 0.0441 ± 0.0005, *P* = 0.017, Figure 6A). Further, the expression and promoter methylation levels of *UTP6* were analyzed in CRC stem and non-stem cells. The relative UTP6 expression was significantly reduced in CRC stem cells compared to CRC non-stem cells (GSE14773: 10.33 ± 0.13 vs. 10.80 ± 0.14; *P* = 0.04; GSE24747: 3.44 ± 0.09 and 3.81 ± 0.00, *P* = 0.03; Figures 6C,D). We analyzed promoter methylation in CRC stem cells in the GSE104271 dataset. The results demonstrated that the UTP6 promoter was hypermethylated at cg10893370 (stem cell vs. non-stem cell: 0.06 ± 0.00 vs. 0.09 ± 0.00, *P* = 0.008, Figure 6B) and cg13453082 (0.05 ± 0.01 vs. 0.08 ± 0.00, *P* = 0.03, Figure 6B) sites in CRC stem cells.

### GSEA and Co-expression Gene Analysis

**FIGURE 7:**
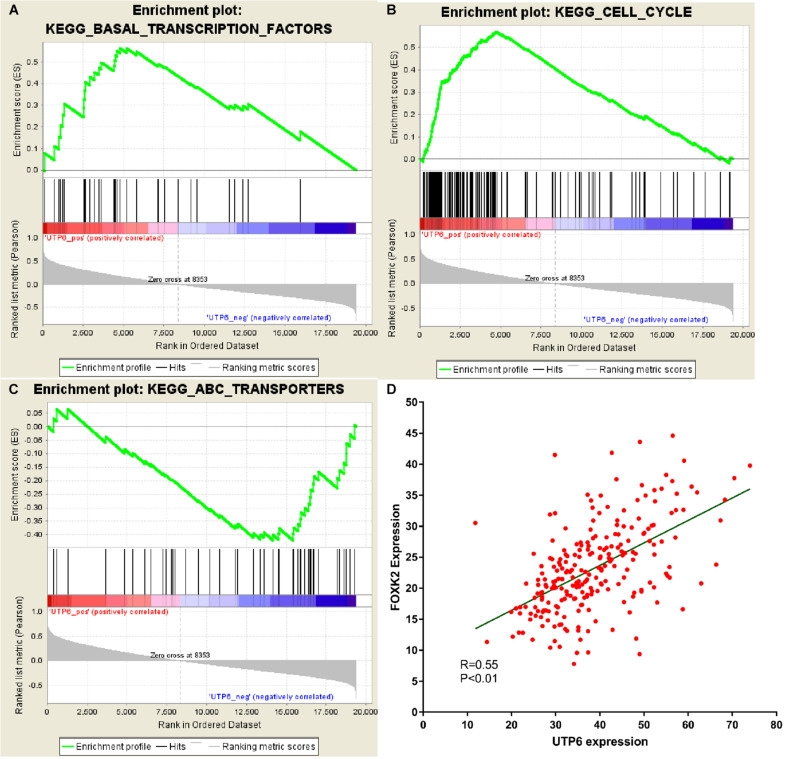
GSEA using our data and co-expression analysis between UTP6 and FOXK2 in the TCGA colon tumor dataset. **(A)** Transcription factors pathway. **(B)** Cell cycle. **(C)** ABC transportanters. **(D)** Co-expression analysis between UTP6 and FOXK2 in the TCGA colon tumor dataset.

GSEA was conducted to investigate the potential mechanism of the UTP6-mediated CRT-resistance in CRC. Our data demonstrated that the negatively correlated KEGG pathways were enriched for the ABC transporter signaling pathway (Figures 7A–C). The positively correlated pathways included transcription factor pathways and cell cycle signaling pathways. To further explore the mechanism of the UTP6-mediated CRT response, the genes co-expressed with UTP6 in the TCGA colon tumor data from ULACAN were analyzed. The results demonstrated that FOXK2 was significantly correlated with UTP6 (*r* = 0.55, *P* < 0.01; Figure 7D).

## Discussion

To date, reliable molecular markers for CRT-resistance in LARC patients are still unavailable. In this study, a gene chip array was performed to detect the gene expression in LARC patients who received CRT. Then WGCNA, an advanced methodology for multigene analysis, was conducted to identify the gene co-expression modules associated with CRT resistance. UTP6 was identified and validated as a hub gene correlated with CRT resistance. High UTP6 expression was correlated with better survival in CRC patients. UTP6 was hypermethylated in CRC tissues, especially in the CRC stem cell subpopulation. The underlying mechanism of UTP6 in maintaining CRC stemness might involve transcription factor pathways, especially FOXK2.

Currently, microarray expression profiling has been utilized to screen biomarkers in patients with CRT-resistant rectal cancer. Conventional molecular biology methodology identifies DEGs, but it is difficult to correlate biological information provided by gene names with biological functions independently. WGCNA has emerged as an effective method to discover the relationship between networks, genes, phenotypes, and biological sample information, thus avoiding the shortcomings of the traditional methods (Bakhtiarizadeh et al., 2018; Gao et al., 2016; Magani et al., 2018).

Herein, we performed WGCNA to identify the actual hub gene in our patient cohort. The results demonstrated that the dark turquoise module was chosen as the hub module and enriched for translation, DNA replication, and androgen receptor signaling pathways. Those pathways are involved in multi-drug resistant CRT tumors, indicating that the dark turquoise module was significantly associated with CRT-resistance. As described previously (Liu et al., 2017; Wang et al., 2018), PPI network analysis and clinical trait correlation were performed to screen for the actual hub gene. The results suggested that the UTP6 gene had the highest correlation coefficient. We further analyzed the expression of UTP6 in our previous data and testing data set (GSE35452), which showed that UTP6 was the relevant gene for CRT-sensitivity. ROC analysis showed that UTP6 could effectively discriminate between CRT-resistant and -sensitive cases. To our knowledge, this was the first study to identify and verify UTP6 as an effective new marker for the prediction of CRT response.

Having shown that UTP6 was associated with CRT-resistance and survival in CRC patients, the underlying mechanism remained unclear. Cancer stem cells are widely known as tumor-initiating cells due to stem cell properties. Increasing evidence has shown that cancer stem cells are involved in the resistance to conventional cytotoxic therapies, radio- and chemotherapy (Baumann et al., 2008; Wahab et al., 2017). Herein, we evaluated the relative UTP6 expression in two independent data sets including CRC stem cells and parental cells (GSE14773 and GSE24747). The results demonstrated that the expression of UTP6 was significantly decreased in CRC stem cells compared to CRC non-stem cells.

DNA methylation, a main epigenetic modification in the mammalian genome (Baylin et al., 2001; Herman and Baylin, 2003), may inactivate genes and affect the CRT response (Wilting and Dannenberg, 2012; Ivanova et al., 2013). Several studies have reported that DNA methylation could promote drug or radiation resistance (Su et al., 2011), and thus, could be used to predict treatment response. In the present study, we found hypermethylation in colon cancer tissue and the cg10893370 and cg13453082 sites located in the UTP6 promoter in CRC stem cells. These results indicated that the hypermethylation of UTP6 may result in a decreased expression of UTP6 in CRC stem cells and that the downregulation UTP6 expression in cancer stem cells may be due to hypermethylation of the promoter sites. Several studies have reported that DNA methylation could affect gene expression in the transcriptome, which is involved in CRT-resistance in various cancer stem cells (Kurscheid et al., 2015; Kostopoulou et al., 2018). Importantly, DNA methylation can be reversed by anti-tumor drugs, such as decitabine (Ariazi et al., 2017; Flotho et al., 2018). The findings suggest that the combination of demethylation therapy targeting *UTP6* and cancer stem cells during CRT might be beneficial to rectal cancer patients.

UTP6 (also known as HCA66) is an essential component of the UTPB, a large complex composed of six proteins Utp1/Pwp2, Utp6, Utp12/Dip2, Utp13, Utp18, and Utp21 (Grandi et al., 2002; Krogan et al., 2004). Ferraro et al. (2011) reported that Apaf1 could bind with UTP6 and participate in the regulation of centrosomal microtubule nucleation, spindle assembly, cell migration, and mitochondrial network organization. Meanwhile, UTP6 has been proposed as a promising prognostic indicator in bladder cancer (Shivakumar et al., 2017). A previous study reported that UTPB was the consist of the UTP6, which is an important complex in regulating ribosome synthesis (Hunziker et al., 2016; Zhang et al., 2016). Herein, we found that UTP6 was not only related to the ABC transporter signal pathway, but also transcription factor pathways, which are associated with CRT resistance (Shinto et al., 2014; McCoy et al., 2015). Pearson’s correlation analysis showed that the expression of UTP6 was correlated with that of FOXK2, which is a transcription factor known as an anti-oncogene (Shan et al., 2016; de Moraes et al., 2019). The results indicated that the anti-chemoradiotherapy resistance effect of UTP6 may be related to FOXK2 expression. Unfortunately, the precise mechanism of UTP6 involved in CRT sensitivity in rectal cancer patients is still unclear.

There were several limitations to our study. Although the underlying mechanism of UTP6 was explored by bioinformatics analysis, the results warrant further confirmation by *in vitro* or *in vivo* experiments. To our knowledge, this was the first study to explore CRT-resistance-related genes through WGCNA using a patient cohort with complete clinicopathological and follow-up data. The present study provided insight into the role of UTP6 in CRT-sensitive in rectal cancer patients.

## Conclusion

Through WGCNA, UTP6 was identified and validated as a hub gene and a valid novel predictor for CRT-sensitivity. High expression of UTP6 associated with better survival in LARC patients. UTP6 was hypermethylated in CRC tissues, especially in CRC stem cells subpopulation. GSEA demonstrated that the mechanism underlying UTP6 maintains CRC stemness might be involved in the transcription factors pathway especially FOXK2. Further research is needed to confirm the above findings.

## Data Availability Statement

The datasets presented in this study can be found in online repositories. The names of the repository/repositories and accession number(s) can be found below: https://www.ncbi.nlm.nih.gov/geo/, GSE145037.

## Ethics Statement

Studies relative to humans in this article were approved by the Ethics Committee of The Fujian Medical University Union Hospital. The patients/participants provided their written informed consent to participate in this study. Written informed consent was obtained from the individual(s) for the publication of any potentially identifiable images or data included in this article.

## Author Contributions

YZ, QG, YW, GG, and XL designed and performed the experiments, analyzed the data, and wrote the manuscript. YP, JZ, and YY performed the experiments. All authors read and approved the final manuscript.

## Conflict of Interest

The authors declare that the research was conducted in the absence of any commercial or financial relationships that could be construed as a potential conflict of interest.

## Publisher’s Note

All claims expressed in this article are solely those of the authors and do not necessarily represent those of their affiliated organizations, or those of the publisher, the editors and the reviewers. Any product that may be evaluated in this article, or claim that may be made by its manufacturer, is not guaranteed or endorsed by the publisher.

## Footnotes

1
https://www.ncbi.nlm.nih.gov/geo/

2
http://ualcan.path.uab.edu/

3
http://r2.amc.nl

4
http://molpath.charite.de/cutoff/

## Supplementary Material

Supplementary Figure 1 Validation of FTSJ3. **(A)** In our data (6.21 ± 0.32 vs. 5.75 ± 0.47, *P* < 0.01) and **(B)** GSE35452 (0.11 ± 0.40 vs. −0.05 ± 0.53, *P* = 0.246). **(C)** Person analysis between the AJCC TRG grade and FTSJ3 expression. ROC curves and AUC statistics to evaluate the predictive efficiency of the FTSJ3 in our data and external data to distinguish CRT-resistance from CRT−sensitive CRC cases from **(D)** our data and **(E)** GSE35452. The disease-free survival **(F)** and overall survival **(G)** between low and high expression of FTSJ3.

Supplementary Figure 2 The result of Cut-off Finder. Plot of the OR of the UTP6 **(A)** and FTSJ3 **(D)** expression and the optimal cutoff value. And the optimal cutoff value is compared to the gene expression. ROC curves and AUC analysis to evaluate the predictive efficiency of the optimal cutoff of the UTP6 **(B)** and FTSJ3 **(E)** expression. The classification using UTP6 **(C)** and FTSJ3 **(F)** expression and patients’ DFS status. And the optimal cutoff value is compared to the gene expression. OR, odds ratio; DFS, disease-free survival.

## Abbreviations

WGCNA: weighted gene co-expression network analysis
CRT: chemoradiotherapy
CRC: colorectal cancer
LARC: local advanced rectal cancer
GEO: Gene Expression Omnibus
TCGA: the cancer genome atlas
PPI: protein–protein interaction
GSEA: Gene set enrichment analysis
ROC: receiver operating characteristic curve.
