# Supplementary material for: Hypermethylation and Downregulation of UTP6 Are Associated With Stemness Properties, Chemoradiotherapy Resistance, and Prognosis in Rectal Cancer: A Co-expression Network Analysis
Source: Front Cell Dev Biol. 2021 Aug 18;9:607782. doi: 10.3389/fcell.2021.607782 (PMC8416280; doi:10.3389/fcell.2021.607782)
Supplement: Supplementary file 1 [file Table_1.docx]

**Supplementary Table 1** Clinicopathological details of 31 LARC patients

| **No** | **Age（yrs）** | **Sex** | **Pre-CRT cTNM** | **CRT- regimen** | **AJCC-TRG** | **Pathology stage** | **UTP6 expression** | **FTSJ3 expression** | **Recurrence** | **Disease-free survival time(months)** | **Death** | **Overall survival time(months)** |
| --- | --- | --- | --- | --- | --- | --- | --- | --- | --- | --- | --- | --- |
| 1 | 34 | M | cT4N+Mx | 50Gy+XELOX | 2 | ypT3N1Mx | 6.39 | 6.39 | No | 46.63 | No | 58.63 |
| 2 | 66 | F | cT3N+Mx | 50Gy+XELOX | 0 | pCR | 6.60 | 5.92 | No | 46.23 | No | 58.23 |
| 3 | 69 | M | cT3N+Mx | 50Gy+XELOX | 1 | ypT3N0Mx | 6.55 | 6.41 | No | 43.17 | No | 55.17 |
| 4 | 65 | M | cT3N+Mx | 50Gy+XELOX | 0 | pCR | 6.26 | 6.03 | No | 45.83 | No | 57.83 |
| 5 | 72 | M | cT3N+Mx | 50Gy+XELOX | 1 | ypT1N0Mx | 6.13 | 6.54 | No | 42.5 | No | 54.5 |
| 6 | 64 | M | cT4N+Mx | 50Gy+XELOX | 2 | ypT3N0Mx | 5.36 | 4.97 | No | 42.17 | No | 54.17 |
| 7 | 53 | M | cT4N+Mx | 50Gy+XELOX | 2 | ypT3N1Mx | 6.14 | 5.20 | Yes | 18.1 | Yes | 21.1 |
| 8 | 60 | M | cT4N+Mx | 50Gy+XELOX | 2 | ypT3N1Mx | 5.97 | 5.31 | Yes | 30.57 | No | 54.57 |
| 9 | 44 | F | cT3N+Mx | 50Gy+XELOX | 0 | pCR | 6.68 | 6.43 | No | 45.5 | No | 57.5 |
| 10 | 53 | M | cT3N-Mx | 50Gy+XELOX | 0 | pCR | 6.96 | 6.24 | No | 44.3 | No | 56.3 |
| 11 | 58 | F | cT3N+Mx | 50Gy+XELOX | 0 | pCR | 6.33 | 6.29 | No | 43.9 | No | 55.9 |
| 12 | 44 | F | cT3N+Mx | 50Gy+XELOX | 1 | ypT2N1Mx | 5.62 | 5.57 | Yes | 31.4 | No | 55.4 |
| 13 | 41 | F | cT4N+Mx | 50Gy+XELOX | 3 | ypT4N1M1 | 5.94 | 5.90 | Yes | 0 | Yes | 3.8 |
| 14 | 52 | F | cT3N+Mx | 50Gy+XELOX | 2 | ypT3N0Mx | 5.83 | 5.63 | Yes | 35.3 | No | 53.8 |
| 15 | 53 | F | cT2N+Mx | 50Gy+XELOX | 2 | ypT0N1Mx | 6.62 | 6.45 | No | 42.17 | No | 54.17 |
| 16 | 48 | M | cT4N+Mx | 50Gy+XELOX | 2 | ypT3N0Mx | 6.18 | 6.06 | No | 41.03 | No | 53.03 |
| 17 | 49 | F | cT4N+Mx | 50Gy+XELOX | 3 | ypT2N1Mx | 6.65 | 5.93 | No | 38.73 | No | 50.73 |
| 18 | 61 | M | cT4N+Mx | 50Gy+XELOX | 1 | ypT3N0Mx | 6.55 | 5.90 | No | 45.5 | No | 57.5 |
| 19 | 63 | M | cT3N+Mx | 50Gy+XELOX | 1 | ypT2N0Mx | 7.17 | 6.80 | No | 42.57 | No | 54.57 |
| 20 | 75 | M | cT3N+Mx | 50Gy+XELOX | 0 | pCR | 6.88 | 6.54 | No | 42.53 | No | 54.53 |
| 21 | 46 | M | cT4N+Mx | 50Gy+XELOX | 0 | pCR | 6.13 | 5.97 | No | 45.27 | No | 57.27 |
| 22 | 60 | F | cT4N+Mx | 50Gy+XELOX | 0 | pCR | 6.49 | 6.20 | No | 42.53 | No | 54.53 |
| 23 | 59 | M | cT3N+Mx | 50Gy+XELOX | 0 | pCR | 5.66 | 6.31 | No | 41.63 | No | 53.63 |
| 24 | 70 | F | cT3N+Mx | 50Gy+XELOX | 1 | ypT3N0Mx | 6.57 | 6.47 | No | 42.1 | No | 54.1 |
| 25 | 58 | F | cT3N+Mx | 50Gy+XELOX | 0 | pCR | 6.72 | 5.71 | No | 40 | No | 52 |
| 26 | 63 | F | cT3N+Mx | 50Gy+XELOX | 1 | ypT3N0Mx | 6.47 | 6.17 | No | 41.03 | No | 53.03 |
| 27 | 68 | F | cT4N+Mx | 50Gy+XELOX | 1 | ypT2N0Mx | 6.90 | 6.19 | No | 40.7 | No | 52.7 |
| 28 | 41 | M | cT4N+Mx | 50Gy+XELOX | 2 | ypT3N0Mx | 5.58 | 5.69 | No | 40.57 | No | 52.57 |
| 29 | 60 | F | cT3N+Mx | 50Gy+XELOX | 1 | ypT3N1Mx | 6.01 | 5.96 | Yes | 28.8 | Yes | 43.4 |
| 30 | 63 | F | cT4N+Mx | 50Gy+XELOX | 1 | ypT2N1Mx | 6.52 | 6.69 | No | 39.4 | No | 51.4 |
| 31 | 73 | M | cT4N+Mx | 50Gy+XELOX | 2 | ypT3N1M1 | 6.11 | 5.83 | Yes | 0 | Yes | 13.4 |

**M**, male; **F**, female;
