# Supplementary figures and images for: Hypermethylation and Downregulation of UTP6 Are Associated With Stemness Properties, Chemoradiotherapy Resistance, and Prognosis in Rectal Cancer: A Co-expression Network Analysis
Source: Front Cell Dev Biol. 2021 Aug 18;9:607782. doi: 10.3389/fcell.2021.607782 (PMC8416280; doi:10.3389/fcell.2021.607782)

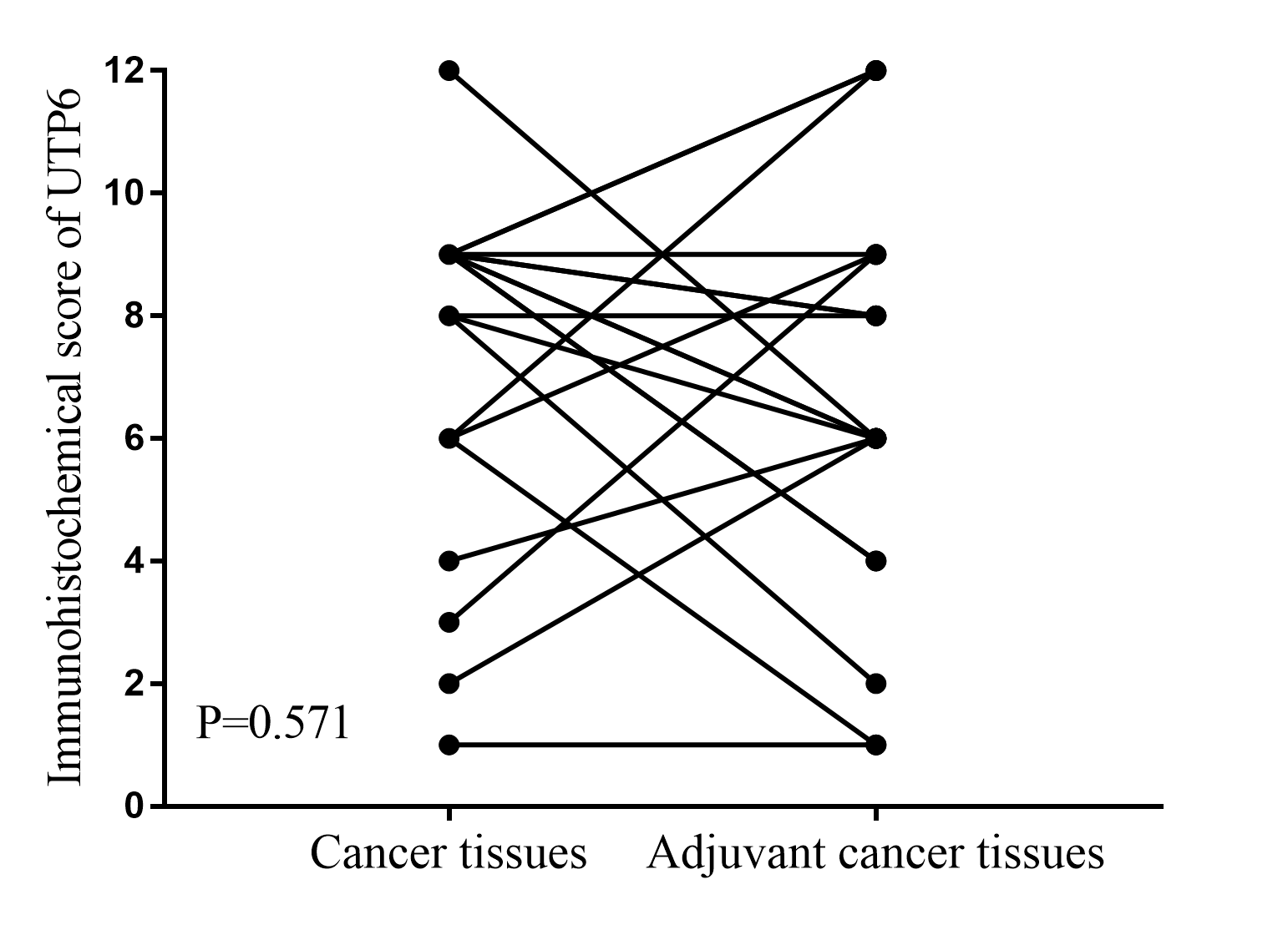

Supplement: Supplementary file 2 [file Image_3.TIF]

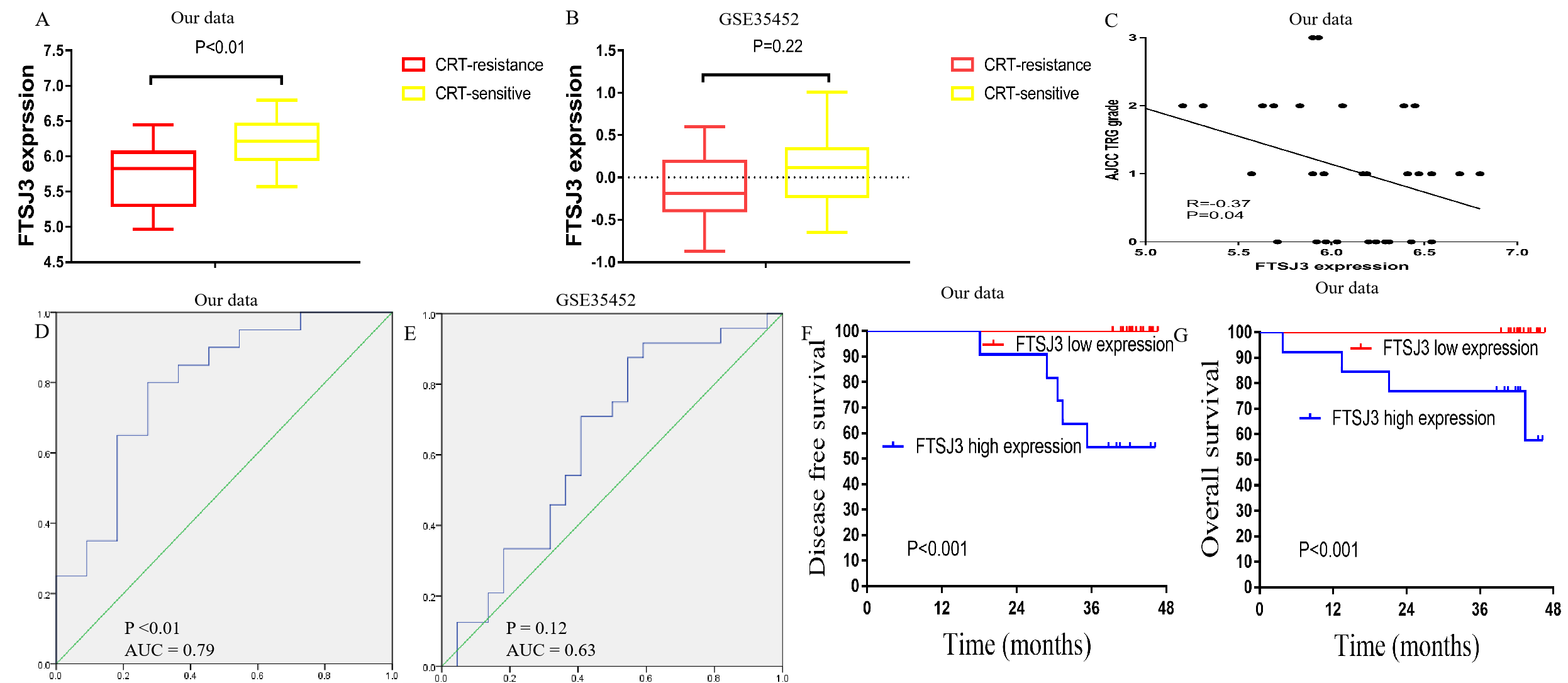

Supplement: Supplementary Figure 1 — Validation of FTSJ3. (A) In our data (6.21 ± 0.32 vs. 5.75 ± 0.47, P < 0.01) and (B) GSE35452 (0.11 ± 0.40 vs. −0.05 ± 0.53, P = 0.246). (C) Person analysis between the AJCC TRG grade and FTSJ3 expression. ROC curves and AUC statistics to evaluate the predictive efficiency of the FTSJ3 in our data and external data to distinguish CRT-resistance from CRT−sensitive CRC cases from (D) our data and (E) GSE35452. The disease-free survival (F) and overall survival (G) between low and high expression of FTSJ3. [file Image_1.TIF]

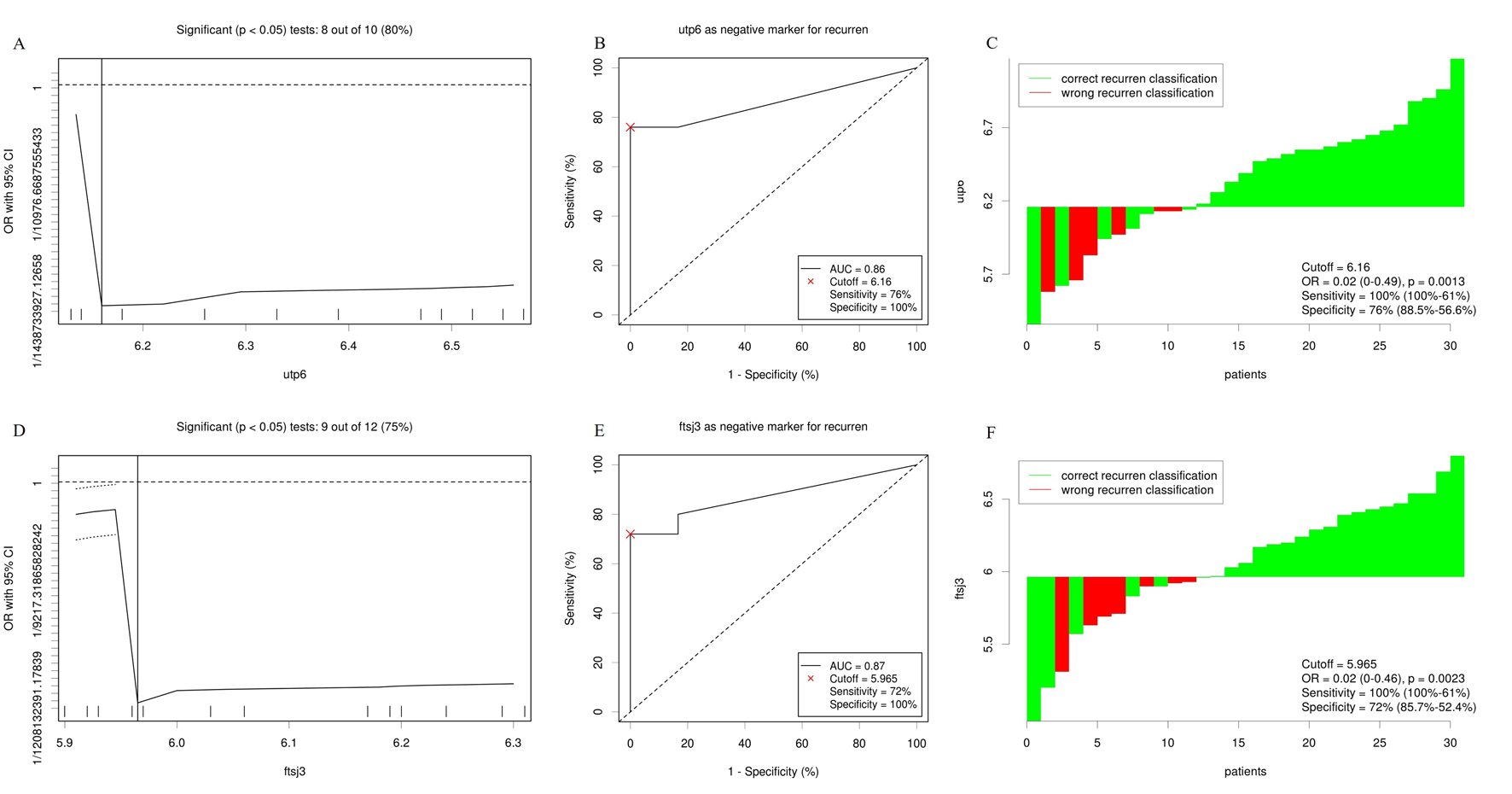

Supplement: Supplementary Figure 2 — The result of Cut-off Finder. Plot of the OR of the UTP6 (A) and FTSJ3 (D) expression and the optimal cutoff value. And the optimal cutoff value is compared to the gene expression. ROC curves and AUC analysis to evaluate the predictive efficiency of the optimal cutoff of the UTP6 (B) and FTSJ3 (E) expression. The classification using UTP6 (C) and FTSJ3 (F) expression and patients’ DFS status. And the optimal cutoff value is compared to the gene expression. OR, odds ratio; DFS, disease-free survival. [file Image_2.TIF]
